# ^99m^Tc-macroaggregated albumin SPECT/CT predictive dosimetry and dose-response relationship in uveal melanoma liver metastases treated with first-line selective internal radiation therapy

**DOI:** 10.1038/s41598-023-39994-7

**Published:** 2023-08-12

**Authors:** Flavian Tabotta, Silvano Gnesin, Vincent Dunet, Alexandre Ponti, Antonia Digklia, Sarah Boughdad, Niklaus Schaefer, John O. Prior, Nicolas Villard, Georgia Tsoumakidou, Alban Denys, Rafael Duran

**Affiliations:** 1 Department of Radiology and Interventional Radiology, Lausanne University Hospital and University of Lausanne, Lausanne, Switzerland; 2 Institute of Radiation Physics, Lausanne University Hospital and University of Lausanne, Lausanne, Switzerland; 3 Department of Nuclear Medicine and Molecular Imaging, Lausanne University Hospital and University of Lausanne, Lausanne, Switzerland; 4 Department of Medical Oncology, Lausanne University Hospital and University of Lausanne, Lausanne, Switzerland

**Keywords:** Cancer therapy, Metastasis, Cancer

## Abstract

First-line selective internal radiation therapy (SIRT) showed promising outcomes in patients with uveal melanoma liver metastases (UMLM). Patient survival depends on liver’s disease control. SIRT planning is essential and little is known about dosimetry. We investigated whether ^99m^Tc-MAA-SPECT/CT dosimetry could predict absorbed doses (AD) evaluated on ^90^Y-PET/CT and assess the dose–response relationship in UMLM patients treated with first-line SIRT. This IRB-approved, single-center, retrospective analysis (prospectively collected cohort) included 12 patients (median age 63y, range 43–82). Patients underwent MRI/CT, ^18^F-FDG-PET/CT before and 3–6 months post-SIRT, and ^90^Y-PET/CT immediately post-SIRT. Thirty-two target lesions were included. AD estimates in tumor and non-tumor liver were obtained from ^99m^Tc-MAA-SPECT/CT and post-SIRT ^90^Y-PET/CT, and assessed with Lin’s concordance correlation coefficients (*ρ*_c_ and *C*_b_), Pearson’s coefficient correlation (*ρ*), and Bland–Altman analyses (mean difference ± standard deviation; 95% limits-of-agreement (LOA)). Influence of tumor characteristics and microsphere type on AD was analyzed. Tumor response was assessed according to size-based, enhancement-based and metabolic response criteria. Mean target lesion AD was 349 Gy (range 46–1586 Gy). Concordance between ^99m^Tc-MAA-SPECT/CT and ^90^Y-PET/CT tumor dosimetry improved upon dose correction for the recovery coefficient (RC) (*ρ* = 0.725, *ρ*_c_ = 0.703, *C*_b_ = 0.969) with good agreement (mean difference: − 4.93 ± 218.3 Gy, 95%LOA: − 432.8–422.9). Without RC correction, concordance was better for resin microspheres (*ρ* = 0.85, *ρ*_c_ = 0.998, *C*_b_ = 0.849) and agreement was very good between predictive ^99m^Tc-MAA-SPECT/CT and ^90^Y-PET/CT dosimetry (mean difference: − 4.05 ± 55.9 Gy; 95%LOA: − 113.7–105.6). After RC correction, ^99m^Tc-MAA-SPECT/CT dosimetry overestimated AD (− 70.9 ± 158.9 Gy; 95%LOA: − 382.3–240.6). For glass microspheres, concordance markedly improved with RC correction (*ρ* = 0.790, *ρ*_c_ = 0.713, *C*_b_ = 0.903 vs without correction: *ρ* = 0.395, *ρ*_c_ = 0.244, *C*_b_ = 0.617) and ^99m^Tc-MAA-SPECT/CT dosimetry underestimated AD (148.9 ± 267.5 Gy; 95%LOA: − 375.4–673.2). For non-tumor liver, concordance was good between ^99m^Tc-MAA-SPECT/CT and ^90^Y-PET/CT dosimetry (*ρ* = 0.942, *ρ*_c_ = 0.852, *C*_b_ = 0.904). ^99m^Tc-MAA-SPECT/CT slightly overestimated liver AD for resin (3.4 ± 3.4 Gy) and glass (11.5 ± 13.9 Gy) microspheres. Tumor AD was not correlated with baseline or post-SIRT lesion characteristics and no dose–response threshold could be identified. ^99m^Tc-MAA-SPECT/CT dosimetry provides good estimates of AD to tumor and non-tumor liver in UMLM patients treated with first-line SIRT.

## Introduction

Uveal melanoma is a radiosensitive cancer as evidenced by the therapeutic success following radiation therapy of the primary eye tumor. Metastases have a tropism for the liver and the prognosis worsens once hepatic spread occurs. Survival following liver involvement ranges from a few months without treatment to 6–13 months following therapy^1,2^. Thus, disease control in the liver is fundamental.

In this setting, selective internal radiation therapy (SIRT) with Yttrium-90-(^90^Y)-microspheres holds promise to improve the outcomes of uveal melanoma liver metastasis (UMLM) patients. In a salvage setting, SIRT obtained survival outcomes of 3–12 months^3–8^. However, when used as first-line therapy, SIRT achieved a median overall survival (OS) of 18 months^9^.

^99m^Tc-macroaggregated albumin (^99m^Tc-MAA) SPECT/CT aims to predict the intra-hepatic distribution and absorbed doses delivered with ^90^Y-microspheres. The reliability of ^99m^Tc-MAA-SPECT/CT-based predictive dosimetry has been demonstrated in several liver tumors such as hepatocellular carcinoma (HCC), and can be assessed post-SIRT by ^90^Y-PET/CT dosimetry^10–12^.

A dose–response relationship has been demonstrated for several tumor types such as HCC, colorectal liver metastases and cholangiocarcinoma^13–17^. However, knowledge about dosimetric data for UMLM is scarce.

The aim of this study was to compare ^99m^Tc-MAA-SPECT/CT-based dosimetry with post-SIRT ^90^Y-time-of-flight PET/CT dosimetry and investigate the dose–response relationship in UMLM patients treated with first-line SIRT. To our knowledge, these topics were not previously assessed in UMLM.

## Material and methods

This single-institution, retrospective analysis of a prospectively collected patient cohort was approved by the Institutional Review Board. Informed consent was waived by the Commission cantonale d'Éthique de la Recherche sur l'être humain du canton de Vaud (CER-VD #2017-01735).

### Patient population and assessment

Thirty-five consecutive patients with UMLM were treated with SIRT between 2012 and 2020. Patients were discussed in a multidisciplinary tumor board and provided informed consent for the procedure.

Inclusion criteria were: (1) biopsy-proven liver metastases; (2) patients who underwent dynamic contrast-enhanced MRI/CT and ^18^F-FDG-PET/CT before and 3–6 months post-SIRT, and ^90^Y-PET/CT immediately post-SIRT; (3) Eastern Cooperative Oncology Group (ECOG) performance status 0–2; (4) adequate liver (bilirubin ≤ 2 mg/dL), hematologic (granulocyte count ≥ 1.5 × 10^9^/L, platelets ≥ 50 × 10^9^/L)); and renal (creatinine > 2 mg/dL) functions^18^. Twenty-three patients were excluded due to the absence of ^90^Y-PET/CT. Thus, the final study population included 12 patients.

### Simulation angiography and SIRT

Simulation angiography was performed with injection of ^99m^Tc-MAA in the hepatic tumor feeding arteries. Single-photon emission CT with integrated CT (SPECT/CT) was acquired to quantify the targeted tumor volume, tumor-to-liver uptake ratio, any extra-hepatic shunt and for predictive dosimetry planning (partition model). Acquisition and reconstruction parameters of ^99m^Tc-MAA-SPECT/CT are summarized in Supplementary Table 1. In the weeks following the simulation angiography, SIRT (SIR-Spheres®, Sirtex Medical, Australia or TheraSphere®, Boston Scientific, USA) was performed as an outpatient procedure^18,19^.

### Predictive and post-SIRT dosimetry

Pre-SIRT predictive dosimetry was obtained form ^99m^Tc-MAA SPECT/CT acquisitions. The post-SIRT absorbed dose verification (metastases, total tumor volume, target liver and non-tumor volume) was performed on quantitative ^90^Y-PET/CT acquired immediately after ^90^Y-microspheres administration (Discovery 690, GE Healthcare, USA and Biograph Vision 600, Siemens Healthineers, USA)^20^. ^90^Y-PET/CT acquisition required 1 or 2 bed positions depending on the axial dimension of the liver. ^90^Y-activity was measured and corrected for radioactive decay between microspheres administration and images acquisition. We reported PET/CT acquisition and reconstruction information in Supplementary Table 2.

We co-registered ^90^Y-PET/CT (and ^99m^Tc-MAA-SPECT/CT) images with MRI/CT images using PMOD software (PMOD Technologies, Switzerland). In PMOD, volume of interest including metastases, non-tumor and target liver were manually or semi-automatically (using an intensity-based threshold) delineated by a dual-trained nuclear medicine physician/radiologist and a nuclear medicine physicist (FT and SG, each having 7 years of experience). 3D-voxelized ^90^Y-dose maps were computed from the ^99m^Tc-MAA-SPECT and post-treatment ^90^Y-PET images using the assumption of local energy deposition (LED) (i.e., the measured voxel activity contributes to the absorbed dose only in the considered voxel) considering permanent microsphere implantation and pure physical decay^21^.

For both ^99m^Tc-MAA SPECT/CT and ^90^Y-PET/CT, we performed phantom experiments based on a NEMA/IEC NU-2 phantom using same acquisition and reconstruction parameters as in patient studies. From these phantom data, we obtained size-dependent recovery coefficient (RC) to correct for the decrease in signal recovery in lesions due to partial volume effects (Supplementary Tables 1–3 and Supplementary Fig. 1). Using the LED, the phantom validation was restricted to the evaluation of the accuracy of the expected signal recovery in terms of activity.

For both predictive and post-treatment dosimetry, total therapeutic administered activity was partitioned proportionally to the relative voxel intensity to obtain 3D-voxelized activity maps (kBq/mL). Using the LED approach, activity-to-absorbed dose conversion considered a multiplicative factor of 50 Gy/(GBq/kg)^22^. In the obtained predictive and post-therapy dose maps (Gy), we calculated average absorbed doses in tumors corrected for partial-volume effects (i.e., upon application of the appropriate RCs). The RC were assessed for the spherical inserts of the NEMA/IEC phantom, and they were obtained by calculating the ratio of the measured and expected mean activity concentration or equivalently the ratio of the measure and expected total activity in each considered spherical insert. In our analysis, the absorbed doses were computed considering LED and consequently average absorbed doses were obtained by multiplication of the VOI activity by the 50 Gy · kg/GBq factor. The RC factors were than applied to the average absorbed dose of each specific lesion in accordance with the estimated lesion mass. This process gives the same results if the RC is applied to the VOI activity before scaling to dose. Specific RC coefficients were generated for the ^99m^Tc-SPECT and ^90^Y-PET image reconstructions. The use of the LED assumption was motivated by the relatively small lesion size that suffered for signal spill out in liver, due to the limited image resolution in addition to the presence of respiratory motion. The alternative use of voxel-S values or Monte Carlo methods would have resulted in an additional spatial broadening of the absorbed dose pattern^23,24^.

### Image analysis

Image analysis was independently performed by two radiologists (RD and FT with 11 and 7 years of experience). Any ambiguity was resolved in consensus. Results were averaged. Readers were blinded to outcomes. Up to four lesions per patient were included. Morphologic measurements (Vue PACS Carestream) comprised the largest overall (Response Evaluation Criteria in Solid Tumors 1.1 (RECIST)) and enhancing (modified RECIST (mRECIST)) tumor diameter and maximum cross-sectional (World Health Organization (WHO)) or enhancing area (European Association for Study of the Liver (EASL))^25–28^. 3D quantitative tumor assessment evaluated the tumor volume (volumetric RECIST (vRECIST)) using a semiautomatic software (Medisys, Philips Research, France)^29^. Patients were classified as responders (complete (CR) or partial response (PR)) or nonresponders (stable (SD) or progressive disease (PD)).

### Metabolic tumor parameters

Tumor and non-tumor liver metabolic activities were measured on baseline and post-SIRT ^18^F-FDG-PET/CT images (AW v3.2, GE Healthcare, USA). Lesions were semi-automatically delimited using a 42% threshold of the maximum voxel value. Metabolic tumor parameters quantified included total lesion glycolysis (TLG, g/mL · cm^3^) as well as with standard uptake values body weighted (SUVbw, g/mL) maximum, mean and peak (i.e. mean value in 1 mL sphere around the “hottest” voxel). Volumetric lesions radiation dosimetry was based on compartment model. Metabolic response was assessed using EORTC criteria^30^.

### Statistical analysis

Statistical analysis was performed using STATA 16.0 (StataCorp, USA). Data were summarized using descriptive statistics (count and frequency for categorical variables and mean/median range for continuous variables). Kruskal–Wallis test was used to compare measurements before and after SIRT as well as ^18^F-FDG positive/negative lesions. Agreement between ^99m^Tc-MAA-SPECT/CT and ^90^Y-PET/CT doses was assessed with the Lin’s concordance correlation coefficients (*ρ*_c_ and *C*_b_), Pearson’s correlation coefficient (*ρ*), and Bland–Altman analyses (mean difference and 95% limits-of-agreement [LOA]^31^). ^99m^TC-MAA-predicted to actual ^90^Y-dose ratio was also analyzed. Doses were measured with and without RC correction. We looked for a correlation between pre- or post-SIRT lesion characteristics or response categorization and doses (predicted, delivered and predicted to actual dose ratio) with the Spearman’s rank order correlation (rho). A *P* value < 0.05 was considered significant. For multiple comparisons, the significance level was corrected using the Bonferroni method (indicated in the Tables’ footnotes). The response distribution regarding investigated response criteria/classification was correlated to the absorbed dose (patient and lesion levels). Kaplan–Meier method with the log-rank test was used to determine OS (day of SIRT until death/last follow-up).

### Ethics approval

This study was performed in line with the principles of the Declaration of Helsinki. Approval was granted by the Commission cantonale d'Éthique de la Recherche sur l'être humain du canton de Vaud (CER-VD #2017-01735).

### Consent to participate

This study was performed in line with the principles of the Declaration of Helsinki. Approval was granted by the Commission cantonale d'Éthique de la Recherche sur l'être humain du canton de Vaud (CER-VD #2017-01735).

## Results

### Patient data

Patient and tumor characteristics are summarized in Tables 1 and 2. Patient cohort was composed of 7 females and 5 males. Mean patient age was 63 years. Most patients were ECOG 0. Thirty-two target lesions were included, of which twenty-four were ^18^F-FDG-positive and eight were ^18^F-FDG-negative. Baseline target tumor characteristics were similar between ^18^F-FDG negative/positive lesions and in patients treated with resin or glass-microspheres. The median tumor diameter and volume were 1.8 cm (range, 0.8–5.7) and 4.8 cm^3^ (range, 0.6–109.7), respectively.

**Table 1:** Patient characteristics.

Characteristic	Value (%)
No. of patients	12 (100)
Sex	
Male	5 (42)
Female	7 (58)
Age	
All patients	62.2 (range, 43–82)
Female	62 (range, 50–73)
Male	62.4 (range, 43–82)
Ethnicity	
White	11 (91.7)
Other	1 (8.3)
ECOG status	
0	11 (91.7)
1	1 (8.3)
Time from diagnosis of UMLM (months)	
Mean	39.2 (95%CI, 16.2–62.2)
Median	26.5 (range, 0–144)
Time from diagnosis of liver metastases to first SIRT (months)	
Mean	5.4 (95%CI, 2–8.8)
Median	3 (range, 1–22)
No. of patients with extrahepatic metastases before SIRT	3 (25)
Post-SIRT systemic therapies	
Chemotherapy	2 (17)
Immunotherapy	4 (33)
Both	5 (42)
Post-SIRT locoregional therapies	
TACE	3 (25)
Thermal ablation	2 (17)
SIRT	2 (17)
TACE + SIRT	1 (8)
Thermal ablation + SIRT	1 (8)

Except where indicated, data represent number of patients and numbers in () are percentages.

**Table 2:** Baseline tumor characteristics.

Characteristic	Value (%)
Liver tumor distribution	
Whole liver	10 (83.3)
Unilobar	2 (16.7)
Liver tumor burden	
Number of target lesions	32 (100)
Liver tumor volume (cm^3^)	
Mean	134.8 (95%CI, 62.6–207)
Median	114 (range, 10–510)
Number of metastases	
0–10	8 (66.7)
≥ 11	4 (33.3)
Largest lesion in cm per patient	
Mean	2.9 (95%CI, 2.05–3.75)
Median	2.2 (range, 1.5–5.7)
Target lesion characteristics	
Diameter (cm)	
Mean	2.1 (95%CI, 1.7–2.5)
Median	1.8 (range, 0.8–5.7)
Surface (cm^2^)	
Mean	4.9 (95%CI, 2.6–7.2)
Median	2.5 (range, 0.5–31.9)
Enhancing diameter (cm)	
Mean	2.1 (95%CI, 1.6–2.6)
Median	1.6 (range, 0.2–5.7)
Enhancing surface (cm^2^)	
Mean	4.8 (95%CI, 1.9–7.7)
Median	1.4 (range, 0.2–40.2)
Tumor volume (cm^3^)	
Mean	11.6 (95%CI, 3.6–19.6)
Median	4.8 (range, 0.6–109.7)
Functional tumor parameters (per target lesion)	
^18^F-FDG positive lesions	24 (75)
^18^F-FDG negative lesions	8 (25)
SUV_bw_ max (g/ml, n = 32)	
Mean	6.3 (95%CI, 5.5–7.1)
Median	6.6 (range, 2.6–10.7)
SUV_bw_ mean (g/ml, n = 32)	
Mean	4.0 (95%CI, 3.6–4.4)
Median	4.2 (range, 1.8–6.4)
SUV_lbm_ peak (g/ml, n = 32)	
Mean	4.8 (95%CI, 4.2–5.5)
Median	5.0 (range, 2.0–8.8)
TLG (g/ml · cm^3^, n = 32)	
Mean	58.6 (95%CI, 9.1–108)
Median	16.6 (range, 5.5–617.4)

Except where indicated, data represent number of patients and numbers in () are percentages.

### Treatment data

Table 3 summarizes SIRT characteristics.

**Table 3:** SIRT characteristics.

Characteristic	Value (%)
No. of SIRT procedures	13 (100)
Number of SIRT procedures per patient	
1	11 (92)
2	1 (8)
Liver treatment	
Whole liver in single session	7 (59)
Whole liver in lobar sessions	1 (8)
Lobar only	4 (33)
Sum of administered activities per patient (GBq)	
Mean	1.7 (95%CI, 1.2–2.2)
Median	1.5 (range, 0.8–4.1)
Mean administered activity per patient (GBq)	
Mean	1.6 (95%CI, 1.1–2.1)
Median	1.4 (range, 0.8–4.1)
Highest administered activity per patient (GBq)	
Mean	1.6 (95%CI, 1.1–2.1)
Median	1.4 (range, 0.8–4.1)
Tumor-to-healthy liver uptake ratio	
Mean	4.5 (95%CI, 3.3–5.7)
Median	3.8 (range, 2.5–10)
Dose to tumor (Gy)	
Total targeted tumor	
Mean	206.1 (95%CI, 167–245)
Median	181 (range, 128–300)
^18^F-FDG positive lesions	
Mean	385 (95%CI, 258–512)
Median	241 (range, 46–1586)
^18^F-FDG negative lesions	
Mean	249 (95%CI, 199–300)
Median	247 (range, 88–513)
Dose to healthy Liver (Gy)	
Mean	53 (95%CI, 41–65)
Median	44 (range, 30–99)
Dose to lungs (Gy)	
Mean	1.5 (95%CI, 1.1–1.7)
Median	1.2 (range, 0.2–3.1)
^90^Y-microspheres	
TheraSphere	4 (33)
SIR-Spheres	8 (67)

Except where indicated, data represent number of patients and numbers in () are percentages.

#### ^99m^Tc-MAA-SPECT/CT ^90^Y predictive dosimetry assessment

**Figure 1:**
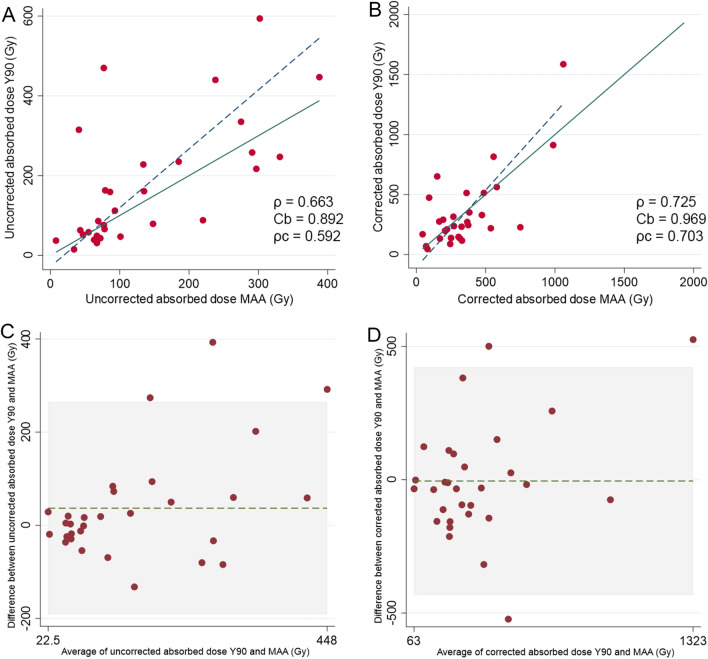
Comparison of absorbed tumor dose between 90Y-predictive dosimetry by 99mTc-MAA-SPECT/CT and post-SIRT dosimetry on 90Y-PET/CT. Plots illustrating Lin’s concordance correlation coefficient between predicted and effective tumor doses in uncorrected (**A**) and corrected (**B**) doses. Dark blue dashed lines represent linear regression fitted with least-squares method and green lines represent perfect concordance. Bland–Altman plot comparing agreement of dose measurements between 90Y-predictive dosimetry by 99mTc-MAA-SPECT/CT and post-SIRT dosimetry without (**C**) and with dose correction (**D**).

There was a good concordance between predicted doses by ^99m^Tc-MAA-SPECT/CT and actual tumor doses measured on ^90^Y-PET/CT, with and without RC correction. However, precision (*ρ*) and accuracy (through the bias coefficient *C*_b_) improved upon dose correction (Fig. 1A–B). Overall, for uncorrected measurements, there was a good agreement with a mean difference of 36.97 Gy between predictive ^99m^Tc-MAA-SPECT/CT dosimetry and ^90^Y-PET/CT dosimetry, and the majority of points were within the 116.1 Gy standard deviation (SD) (95% LOA: − 190.7–264.6) (Fig. 1C). After RC correction of lesion absorbed doses, agreement improved with a mean difference of − 4.93 Gy between predictive ^99m^Tc-MAA-SPECT/CT dosimetry and ^90^Y-PET/CT dosimetry, and the majority of points were within the SD (218.3; 95%LOA: − 432.8–422.9) (Fig. 1D).

Correlation (precision) and agreement of predicted-to-actual dose ratio with and without RC correction are reported in Supplementary Fig. 2.

**Figure 2:**
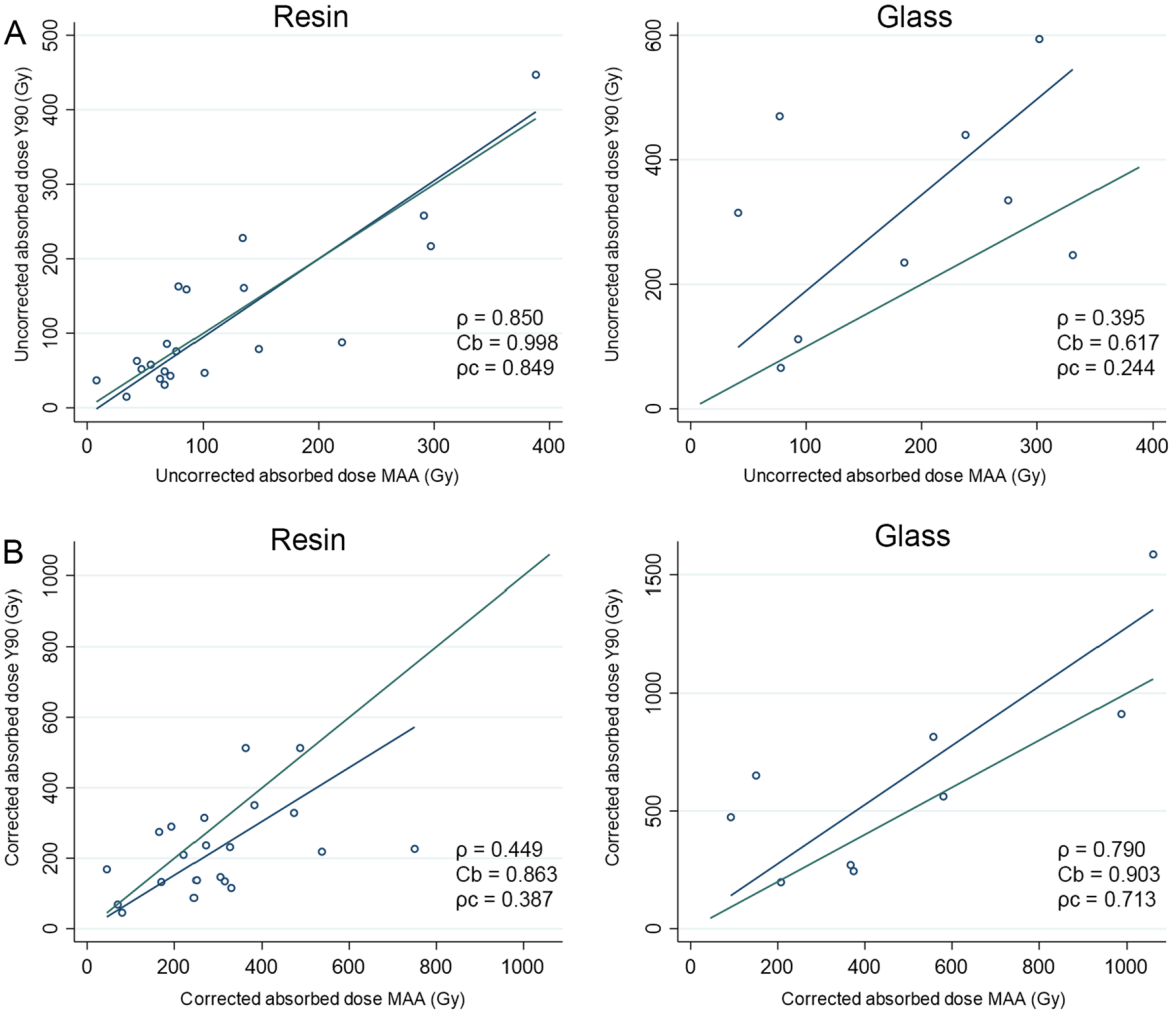
Comparison of absorbed tumor dose between 90Y-predictive dosimetry by 99mTc-MAA-SPECT/CT and post-SIRT dosimetry on 90Y-PET/CT according to the type of microspheres (glass and resin). Dark blue lines represent linear regression fitted with least-squares method and green lines represent perfect concordance.

Without RC correction, concordance was better for resin microspheres (*ρ* = 0.85, *ρ*_c_ = 0.998, and, *C*_b_ = 0.849) and there was a very good agreement between predictive ^99m^Tc-MAA-SPECT/CT dosimetry and ^90^Y-PET/CT dosimetry with a mean difference of − 4.05 Gy (SD: 55.9 Gy; 95%LOA: − 113.7–105.6) (Fig. 2A). After RC correction, ^99m^Tc-MAA-SPECT/CT predictive dosimetry overestimated the delivered dose with resin microspheres (Fig. 2B) and there was a mean difference of − 70.9 Gy (SD: 158.9 Gy; 95%LOA: − 382.3–240.6). With glass microspheres, concordance markedly improved after correction (*ρ* = 0.790, *ρ*_c_ = 0.713, *C*_b_ = 0.903). ^99m^Tc-MAA-SPECT/CT predictive dosimetry underestimated the delivered dose (mean difference: 148.9 ± 267.5 Gy; 95%LOA: − 375.4–673.2); Fig. 2B).

The concordance was good between the predicted-to-delivered absorbed dose ratio without and with RC correction in resin (*ρ* = 0.986, *ρ*_c_ = 0.896, *C*_b_ = 0.909, mean difference − 0.28 ± 0.22, 95%LOA = − 0.69–0.15) and glass (*ρ* = 0.976, *ρ*_c_ = 0.895, *C*_b_ = 0.917, mean difference − 0.17 ± 0.11, 95%LOA = − 0.38–0.04) microspheres (Supplementary Fig. 3).

**Figure 3:**
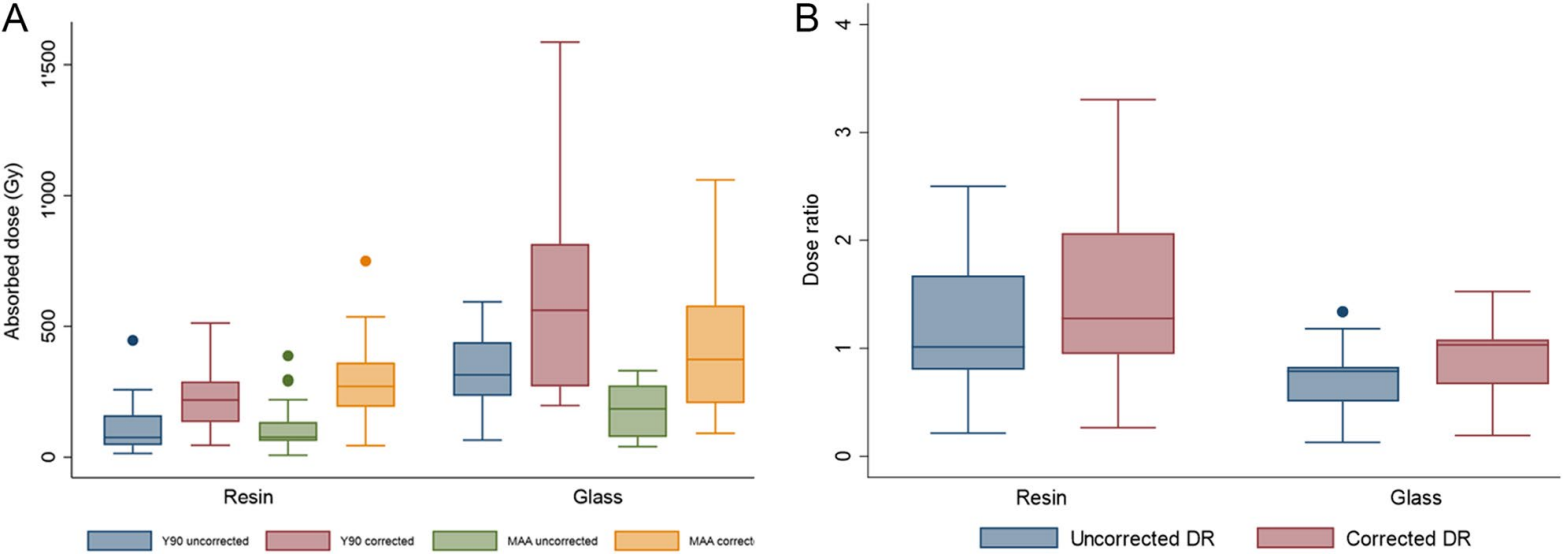
Box plots illustrating the impact of dose correction in absorbed tumor dose between 90Y-predictive dosimetry by 99mTc-MAA-SPECT/CT and post-SIRT dosimetry on 90Y-PET/CT according to the type of microspheres (**A**). Box plots of predicted-to-actual tumor dose ratios (DR) for resin and glass microspheres (**B**).

We obtained a wide range of RC uncorrected and corrected absorbed dose in lesion estimated from the ^99m^Tc-MAA SPECT/CT (range 8–388 and 45–1060 Gy, respectively) and from ^90^Y-PET/CT (range 15–686 and 46–1586 Gy, respectively) (Table 4). Figure 3 illustrates the effect of the RC correction in estimated lesion absorbed dose from ^99m^Tc-MAA SPECT/CT and ^90^Y-PET/CT for resin and glass microspheres.

**Table 4:** ^99m^Tc-MAA SPECT/CT predicted and actual ^90^Y-PET/CT tumor-absorbed doses (after RC correction) for resin and glass microspheres.

	Dose ^99m^Tc-MAA SPECT/CT		Dose ^90^Y-PET/CT		Dose ratio	
	Resin	Glass	Resin	Glass	Resin	Glass
Dose to tumor (Gy)						
Mean	297	486	226	635	1.51	0.87
Median	271	374	219	562	1.28	1.03
Minimum	45	92	46	198	0.27	0.19
Maximum	750	1 060	513	1 586	3.30	1.53
Dose to non-tumor liver (Gy)						
Mean	32	71	29	58	1.14	1.21
Median	29	71	27	61	1.16	1.13
Minimum	20	37	17	35	0.90	1.04
Maximum	53	108	48	75	1.26	1.57

**Figure 4:**
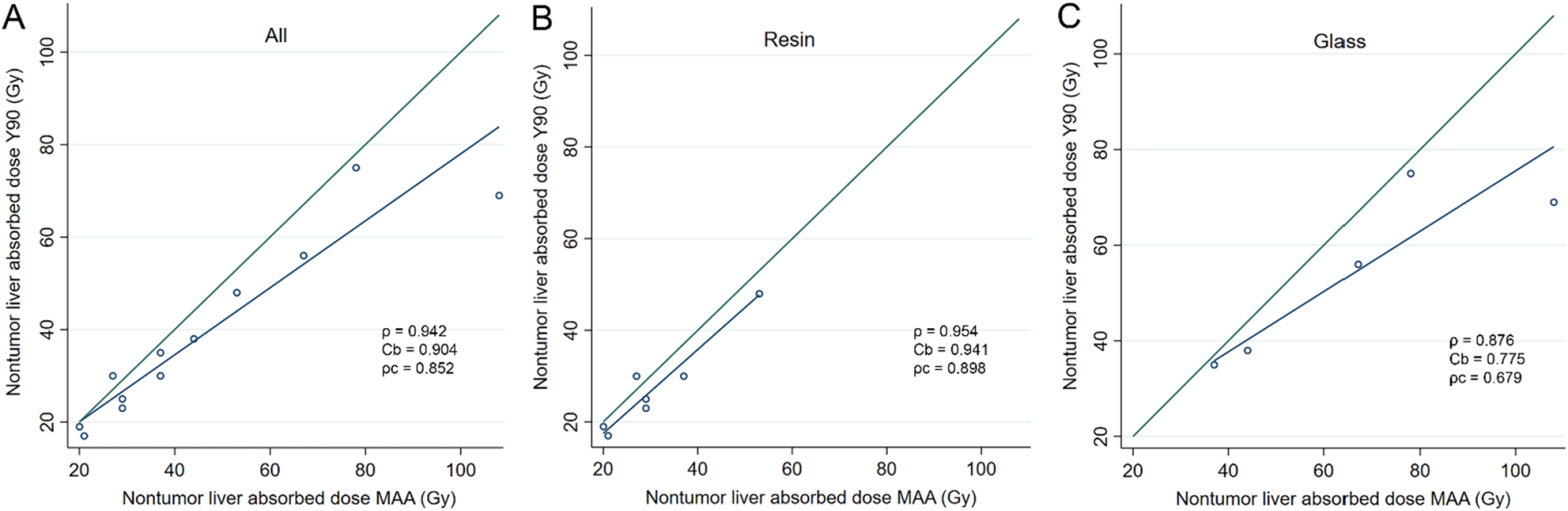
Comparison of absorbed nontumor liver dose between 90Y-predictive dosimetry by 99mTc-MAA-SPECT/CT and post-SIRT dosimetry on 90Y-PET/CT. Plots illustrating Lin’s concordance correlation coefficient for all patients (**A**), resin (**B**) and glass (**C**) microspheres. Dark blue lines represent linear regression fitted with least-squares method and green lines represent perfect concordance.

**Figure 5:**
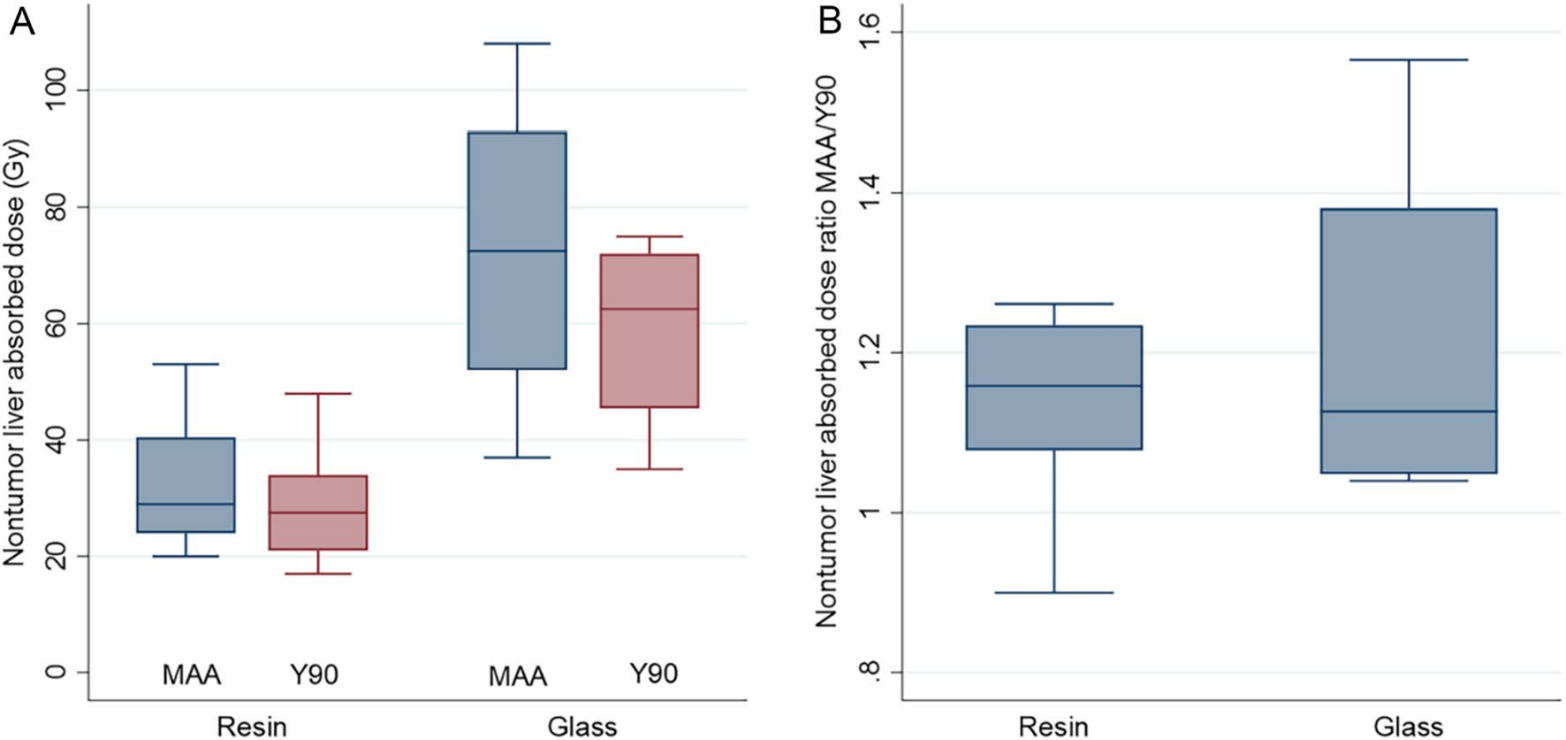
Box plots of absorbed nontumor live dose between 90Y-predictive dosimetry by 99mTc-MAA-SPECT/CT and post-SIRT dosimetry on 90Y-PET/CT according to the type of microspheres (**A**). Box plot of predicted-to-actual nontumor liver dose ratios (DR) for resin and glass microspheres (**B**).

For the non-tumor liver dose (Table 4), there was a good concordance between predicted to post-SIRT absorbed doses (Fig. 4). Figure 5 illustrates ^99m^Tc-MAA SPECT/CT predicted vs. post-SIRT ^90^Y-PET/CT non-tumor liver doses for resin and glass microspheres. ^99m^Tc-MAA SPECT/CT dosimetry slightly overestimated the delivered dose with a mean difference of 3.4 Gy (SD: 3.4 Gy; 95%LOA: − 3.2–10.1) and 11.5 Gy (SD: 13.9 Gy; 95%LOA: − 15.7–38.7) for resin and glass microspheres, respectively, as compared to post-SIRT ^90^Y-PET/CT (quantitative data).

#### Analysis of factors influencing dosimetry

Target lesion absorbed doses were similar between ^18^F-FDG positive and negative lesions (*P* = 0.50). No baseline target lesion characteristics, including lesion volume, and functional tumor parameters (Table 2) were significantly correlated with the ^99m^Tc-MAA SPECT/CT predicted absorbed dose, actually delivered absorbed dose on ^90^Y-PET/CT and predicted-to-post-treatment absorbed dose ratio with or without RC correction and regardless of the lesion’s ^18^F-FDG positive/negative status (all *P* > 0.07).

The absorbed doses estimated in smaller lesions are expected to suffer from large underestimation that can be compensated by application of larger RC. Therefore, the difference between corrected and uncorrected doses on ^99m^Tc-MAA SPECT/CT (rho = − 0.45; *P* = 0.013) and ^90^Y-PET/CT (rho = − 0.5; *P* = 0.0046) was negatively correlated with baseline lesion volume.

Supplementary Table 4 compares target tumor changes post-SIRT in all parameters. After therapy, all lesions’ parameters significantly decreased. Supplementary Table 5 further stratifies this data according to ^18^F-FDG positive/negative status. There was no significant correlation between post-SIRT tumor parameters and ^90^Y-absorbed dose for all lesions and regardless of ^18^F-FDG positive/negative status.

#### Tumor response

Table 5 summarizes tumor response analysis. There was no relationship between absorbed dose and response criteria/classification scales (Table 5), and regardless of the microsphere type (data not shown).

**Table 5:** Tumor response analysis.

^90^Y-absorbed dose according to response criteria -All patients	PD	SD	PR	CR	*P* value
WHO	0	9	3	0	0.08
RECIST 1.1	0	10	2	0	0.39
vRECIST	0	12	0	0	NA
mRECIST	1	6	5	0	0.76
EASL	2	1	9	0	0.91
TLG	1	7	1	2	0.20
SUVmax	1	5	3	2	0.91
SUVmean	1	5	3	2	0.18
SUVpeak	0	6	3	2	0.10

^90^Y-absorbed dose according to response criteria -All lesions	PD	SD	PR	CR	*P* value
WHO	2	21	9	–	0.26
RECIST 1.1	1	26	5	–	0.68
vRECIST	1	27	4	–	0.24
mRECIST	3	15	10	3	0.93
EASL	5	6	17	3	0.51
TLG	4	15	3	6	0.61
SUVmax	2	12	7	6	0.75
SUVmean	4	18	–	6	0.51
SUVpeak	4	17	1	6	0.50

^90^Y-absorbed dose according to response criteria—^18^F-FDG positive lesions	PD	SD	PR	CR	*P* value
WHO	2	14	8	–	0.23
RECIST 1.1	1	18	5	–	0.54
vRECIST	1	20	3	–	0.17
mRECIST	2	13	6	3	0.64
EASL	4	5	12	3	0.18
TLG	3	12	3	6	0.77
SUVmax	2	9	7	6	0.82
SUVmean	3	15	–	6	0.55
SUVpeak	3	14	1	6	0.60

^90^Y-absorbed dose according to response criteria—^18^F-FDG negative lesions	PD	SD	PR	CR	*P* value
WHO	–	7	1	–	0.13
RECIST 1.1					NA
vRECIST	–	7	1	–	0.13
mRECIST	1	2	4	–	0.49
EASL	1	1	5	–	0.60
TLG	1	3	–	–	0.65
SUVmax	1	3	–	–	0.65
SUVmean	1	3	–	–	0.65
SUVpeak	1	3	–	–	0.65

### Survival analysis

Two patients were still alive at the time of the analysis and 10 had died. Median OS after diagnosis of UMLM and after the first SIRT was 26.5 months (95% confidence interval (95%CI), 13.5–50.5) and 23.2 months (95%CI, 16.6–32.8), respectively. Median OS was 18.5 months (95%CI, 13.3–48.5) and 23 months (95%CI, 11.3–29.3) for patients treated by resin vs. glass microspheres, respectively (*P* = 0.57). The median overall and hepatic progression-free survival after the first SIRT were 4 months (95%CI, 2.6–7.4) and 8 months (95%CI, 5.5–14.5), respectively.

## Discussion

Uveal melanoma responds well to local eye irradiation. Upon development of liver metastases, the prognosis is dismal. Thus, SIRT is a promising treatment option for UMLM patients and a better understanding of dosimetry is necessary to further improve outcomes.

^99m^Tc-MAA-particles are widely used as surrogate of ^90^Y-microspheres distribution, with good dosimetric prediction in HCC and other livers tumors^10,11,32–34^. However, different distribution patterns may be observed between ^99m^Tc-MAA and ^90^Y-particles likely due to differences in morphology, size range, number and density^35^.

In our study, we showed that ^99m^Tc-MAA-SPECT/CT dosimetry accurately predicted the delivered dose assessed on post-SIRT ^90^Y-PET/CT in UMLM patients treated with first-line SIRT. After RC correction of measurements, the concordance between predicted-to-actual tumor-absorbed doses improved, with higher precision and accuracy (*ρ* = 0.725, *ρ*_c_ = 0.703, *C*_b_ = 0.969). The agreement also improved upon dose correction, with a mean difference of − 4.93 Gy between predictive ^99m^Tc-MAA-SPECT/CT and ^90^Y-PET/CT dosimetry.

Due to the inherent physical properties of the acquisition device (detector sensitivity and spatial resolution), the absorbed dose of small lesions is underestimated with both ^99m^Tc-MAA-SPECT/CT^36,37^ and post-SIRT ^90^Y-PET/CT^38^ dosimetry. Similarly, we found that the difference between corrected and uncorrected doses either on ^99m^Tc-MAA-SPECT/CT or ^90^Y-PET/CT was negatively correlated with baseline lesion volume, and the correction for small lesions had a major impact. A RC factor is therefore necessary to correct the dose for a more accurate estimation. Indeed, the median of all corrected predicted-to-actual dose ratios was 1.08 (8% overestimation) compared to 0.88 (12% underestimation) without correction.

We found that predictive ^99m^Tc-MAA-SPECT/CT dosimetry slightly overestimated the tumor-absorbed dose with resin microspheres and underestimated it with glass microspheres. Concordance was better before RC correction for resin microspheres and after correction for glass microspheres. These results are in agreement with previously published data for HCC, cholangiocarcinoma and other liver metastases^10,11,32,33,39,40^.

^99m^Tc-MAA-SPECT/CT provided an overall good estimation of the delivered dose to the non-tumor liver, and the correlation was better with resin than with glass microspheres. The non-tumor liver received a higher dose with glass than with resin microspheres, as observed with targeted lesions. ^99m^Tc-MAA-SPECT/CT predictive dosimetry slightly overestimated the delivered dose to the target lesions for both microspheres-type when compared to post-SIRT ^90^Y-PET/CT dosimetry. One potential explanation is that the magnitude of partial volume effect in ^99m^Tc-MAA-SPECT/CT is higher with some activity that spills out from the tumor into the surrounding non-tumor liver. On SPECT/CT, small lesions seem to accumulate less ^99m^Tc-MAA, which could lead to a suboptimal ratio between tumor/non-tumor liver volumes. Another contributing factors could be a more targeted treatment delivery with ^90^Y-microspheres when compared to ^99m^Tc-MAA-particles and the higher spatial resolution of ^90^Y-PET/CT.

We thoroughly analyzed factors influencing dosimetry using quantitative volumetric, enhancement and metabolic parameters. Tumor-absorbed dose was not correlated with baseline or post-SIRT lesions characteristics, including lesion volume, and regardless of the ^18^F-FDG status, although these parameters decreased significantly post-SIRT. Few studies investigated this topic. The percentage of enhancing tumor volume correlated with greater ^90^Y-uptake in the tumor in a heterogeneous cohort of HCC and other liver tumors^41^.

Many studies investigated the dose cut-off values to obtain a tumor response. A recent systematic review of current evidence on dose–response relation showed that response could be obtained with tumor dose thresholds such as 100–250 Gy for HCC and 40–60 Gy for colorectal cancer metastases, with lower doses cut-off for resin microspheres^42^. These wide ranges of dose thresholds can be explained by studies heterogeneity in terms of choice for specific tumor response criteria and timing for response assessment, as well as methods for defining thresholds. Moreover, 17/37 studies included in the analysis did not evaluate post-SIRT dose but an estimation of the absorbed dose was done based on baseline ^99m^Tc-MAA-SPECT/CT^42^. In our study, we observed a wide range of tumor-absorbed dose on ^99m^Tc-MAA-SPECT/CT predictive and ^90^Y-PET/CT dosimetry. Tumor-absorbed dose was not correlated with response despite in-depth analysis of existing size-based, enhancement-based and metabolic response criteria/classifications and we could not identify a dose threshold of response. The majority of targeted lesions (n = 22/32) received > 200 Gy, thus determination of a dose–response threshold may be challenging in this setting. Moreover, uveal melanoma is a radiosensitive cancer and UMLM might not need as high-absorbed doses as other cancer types. Indeed, we observed similar response rate across lesions receiving high and low ^90^Y-doses with low absorbed doses still achieving a response in many targeted tumors. This observation suggests the hypothesis that lower ^90^Y-microspheres activity might be administered with similar efficacy and potentially allows to preserve liver function for repeat SIRT depending on the course of the disease. Of note, our patient cohort treated with first-line SIRT had somehow limited tumor burden. Thus, our results may not be applicable to patient with more advanced liver disease. Further dosimetric research is needed.

The strengths of this work include multi-stratification of results according to type of ^90^Y-microspheres, and a thorough investigation of the dose–response relationship using sized-based and enhancement-based tumor response criteria together with quantitative metabolic parameters. There were limitations. First, is the retrospective design. However, we used a prospectively collected database that limits selection bias. Second, our cohort is relatively small. However, uveal melanoma is a rare disease. Moreover, inclusion of patients treated by first-line SIRT who had available multimodal imaging and ^90^Y-PET/CT allowing such analyses is challenging. Furthermore, our sample size is comparable to previously published data^3,5,6,8^. Third, tumor-absorbed dose and response (in particular for lesions with high signal intensities on precontrast T1-weighted images) determination may be more difficult in small lesions such as those in our cohort. Similarly, the RC correction causes a greater heterogeneity in the distribution of the smaller lesions’ doses, as this corrective factor is directly inversely proportional to the tumor size. Probably in larger tumors a more accurate quantification would be possible. Indeed, we expect that activity and absorbed dose estimates in tumors of larger size, hence suffering of lower partial volume effect, and being easier to register, would be more accurate than for small lesions, in both emission image modalities. Further studies on larger lesions are needed. Considering the quantification, a certain fraction of the PET/SPECT emission signal was present out of the liver VOI delineated in the registered CT. Such a signal spill-out due to the respiratory movement was mainly present in the lower right lung region. Because of the activity map generation via the redistribution of the administered activity proportionally to the voxel intensity, the resulting absorbed dose in the liver might suffer for a bias proportional to the entity of the specific patient spill-out. Nevertheless, the spill-out from the liver was always < 5% of the total emission signal. Thus, the possible overestimation of the absorbed doses in the liver is expected within the same level. Another possible limitation is the use of LED in our study instead of employing dose-point-kernel or Monte Carlo methods. This point is questionable. In fact, the spatial emission signal present in the reconstructed images (SPECT or PET) is convoluted with the finite image resolution (~ 1 cm in SPECT and ~ 0.5 cm in PET) and additionally blurred by the presence of breathing movement that is known to impact the liver (amplitude of 1–2 cm^43^). If these confounding factors could be sufficiently reduced, the dose-point-kernel and Monte Carlo estimations are expected to provide superior performances in terms of spatial energy deposition and hence absorbed accuracy. In the clinical implementation of pre- and post-SIRT imaging, the level of spatial blurring of the signal is comparable or even superior to the average range of the beta particle in biological tissues. Thus, no clear advantage of Monte Carlo and dose-point-kernel methods over LED is expected^21,22^. Another possible source of quantitative bias can arise from different scatter corrections applied to both ^99m^Tc-MAA-SPECT and ^90^Y-PET reconstructions obtained from four different devices. For the two SPECT devices the scatter correction was based on a low energy scatter window. Both PET devices implemented an absolute scatter modelling. Finally, we did not consider any dose volume histogram (DVH) analysis. Such type of analysis could be meaningful for the total liver and perfused liver VOIs, but, because of the relatively small lesion size, the value of the information obtained from a DHV analysis in lesion would be doubtful.

In conclusion, we demonstrated that ^99m^Tc-MAA-SPECT/CT dosimetry accurately predicts the delivered dose to both tumor and non-tumor liver in UMLM patients undergoing first-line SIRT. Delivered dose were overestimated by ^99m^Tc-MAA-SPECT/CT dosimetry with resin microspheres and underestimated with glass microspheres. Interestingly, tumor response was not correlated with the absorbed dose and no dose threshold could be identified. Our results suggest that, in patient with low tumor burden and small lesions such as in our study, similar outcomes might be reached with administration of lower ^90^Y-activities potentially allowing to repeat SIRT, if needed. Further investigations are needed to confirm this hypothesis.

## Key points

### Question

Can ^99m^Tc-MAA-SPECT/CT dosimetry predict absorbed doses evaluated on ^90^Y-PET/CT in patients uveal melanoma liver metastases treated with first-line SIRT?

### Pertinent findings

^99m^Tc-MAA-SPECT/CT dosimetry accurately predicts the delivered dose to both tumor and non-tumor liver. Predictive dosimetry tends to overestimate delivered dose with resin microspheres and underestimated it with glass microspheres. Tumor response was not correlated with the absorbed dose, with many targeted tumors that received low absorbed doses still achieving a response.

### Implications for patient care

^99m^Tc-MAA-SPECT/CT accurately predicts the absorbed doses in patients with uveal melanoma liver metastases treated with first-line SIRT. In patients with low tumor burden and small lesions, a lower ^90^Y-activity might be administered with similar efficacy. Further research is needed to investigate this hypothesis.

### Supplementary Information

Supplementary Information.

## Data Availability

The datasets generated during and/or analysed during the current study are available from the corresponding author on reasonable request.
