# Supplementary material for: 99mTc-macroaggregated albumin SPECT/CT predictive dosimetry and dose-response relationship in uveal melanoma liver metastases treated with first-line selective internal radiation therapy
Source: Sci Rep. 2023 Aug 12;13:13118. doi: 10.1038/s41598-023-39994-7 (PMC10423257; doi:10.1038/s41598-023-39994-7)
Supplement: Supplementary file 1 — Supplementary Information. [file 41598_2023_39994_MOESM1_ESM.pdf]

SUPPLEMENTARY MARTERIAL

Supplementary TABLE 1: Main acquisition and reconstruction parameters for treatment planning with ^{99m}Tc-MAA SPECT/CT.

SPECT/CT	Number of frames	Frame duration (s)	Reconstruction algorithm (iterations × subsets)	Post-reconstruction filter	Corrections	Low-energy scatter window	Image matrix	Voxel size (mm)
Discovery 670	120	15	OSEM (2×10)	Butterworth fc =0.48 cycles/cm; ordre 10	AC/SC	(120 keV ± 10%)	128×128	4.42×4.42×4.42
Symbia Intevo	120	15	OSCGM (40×1)	Gaussian FWHM =5 mm	AC/SC/RR	(120 keV ± 10%)	256×256	1.95×1.95×1.95

Supplementary TABLE 2: Main acquisition and reconstruction parameters for treatment verification ^{90}Y-PET/CT. Acquisitions were performed in list-mode. All pertinent image corrections, (normalization, dead time, activity decay, random coincidences, attenuation and scatter corrections) were applied.

PET/CT	Time per bed (min)	Recon. Algorithm (iterations x subsets)	Scatter Model	PSF	TOF	Post-recon. Gaussian filter (FWHM mm)	Image matrix	Voxel size (mm)
Discovery 690	30	OSEM (3×16)	Absolute	no	yes	5	256×256	2.73×2.73×3.27
Biograph Vision 600	15	OSEM (2×5)	Absolute	yes	yes	4	220×220	3.3×3.3×2

In SPECT/CT and PET/CT devices, we obtained recovery coefficients (RC) as a function of size from a phantom experiment in which a NEMA/IEC NU2 test object. For ^{99m}Tc SPECT/CT experiments, the phantom was filled with 5kBq/mL in the main volume (9.7L) while for ^{90}Y PET/CT experiments, the phantom background was filled with 1MBq/mL. Spherical inserts were filled with a 10× and a 5× higher activity concentration in SPECT and PET experiments, respectively. The size and RC values obtained for the six spherical inserts are reported in **Supplementary TABLE 3**. RC data were fitted with a hyperbole function: RC(sph_vol) = C1+C2/(sph_vol +C3) as

displayed in **Supplementary Figure1**. The fit parameters (C1, C2 and C3) were obtained by minimizing a cost function implemented in the fminsearch function of Matlab used at this scope.

The acquired PET phantom data was reconstructed using the same parameters used for patients.

Supplementary TABLE 3: Recovery coefficients and RC fit parameters obtained for the four imaging devices involved in the study.

Sphere size (Ø in mm)	10	13	17	22	28	37	Fit parameters		
Sphere volume (mL)	0.52	1.15	2.57	5.57	11.49	26.51	C1	C2	C3
RC: SPECT Discovery 670	0.15	0.22	0.38	0.44	0.60	0.70	0.994	7.418	8.078
RC: SPECT Symbia Intevo	0.13	0.22	0.38	0.51	0.69	0.80	0.998	5.083	5.248
RC: PET Discovery 690	0.15	0.35	0.41	0.53	0.68	0.81	0.985	4.291	4.643
RC: PET Biograph Vision 600	0.25	0.43	0.61	0.71	0.72	0.84	0.944	1.554	1.682

Supplementary TABLE 4: Changes in target tumor parameters following SIRT.

	**Pre-SIRT Median [IQR]**	**Post-SIRT Median [IQR]**	***p*-value**
Surface	2.5 [1.32-4.36]	1.6 [1.04-3.88]	0.0003
Diameter	1.8 [1.30-2.25]	1.4 [1.15-2.25]	0.0006
Volume	4.8 [1.25-8.56]	2.6 [0.85-5.69]	<0.0001
Enhancing diameter	1.6 [1.20-2.30]	1.3 [0.70-2.00]	0.0004
Enhancing surface	1.4 [0.54-4.40]	0.5 [0.24-1.60]	0.0004
TLG	16.6 [10.2-25.7]	13.5 [4.5-23.5]	0.0025
SUVmax	6.6 [3.78-8.11]	4.6 [3.45-6.94]	0.0017
SUVmean	4.2 [2.88-4.84]	3.3 [2.67-4.12]	0.0041
SUVpeak	5.0 [3.01-5.88]	3.8 [2.84-4.77]	0.0036

Note: significance level: $p_{uncorr}<0.05$, $p_{corr}<0.0045$

Supplementary TABLE 5: Changes in target tumor parameters following SIRT according to the ^{18}F-FDG positive/negative status.

Variables	^{18}F-FDG negative Median [IQR]		*p*-value	^{18}F-FDG positive Median [IQR]		*P*-value
	Pre-SIRT	Post-SIRT		Pre-SIRT	Post-SIRT	
Surface	1.7 [1.00-3.09]	1.3 [0.59-2.16]	0.025	2.8 [1.92-5.00]	1.6 [1.14-4.37]	0.0030
Diameter	1.5 [1.05-1.85]	1.2 [0.95-1.65]	0.035	1.9 [1.50-2.45]	1.5 [1.20-2.35]	0.0048
Volume	1.1 [0.76-2.85]	0.9 [0.41-1.99]	0.069	5.6 [1.69-9.57]	3.6 [1.60-6.83]	0.0002
Enhancing diameter	1.2 [0.90-1.40]	0.9 [0.60-1.20]	0.10	1.9 [1.40-2.35]	1.5 [1.05-2.30]	0.0018
Enhancing surface	0.7 [0.24-1.26]	0.4 [0.14-0.63]	0.051	2.1 [0.58-4.73]	0.5 [0.34-1.82]	0.0018
TLG	13.4 [9.3-19.1]	15.8 [10.2-22.1]	0.72	16.7 [10.9-35.9]	12.8 [1.7-23.5]	0.0033
SUVmax	3.4 [2.87-3.74]	4.3 [3.84-4.51]	0.47	7.5 [6.55-8.58]	5.5 [1.70-7.21]	0.0010
SUVmean	2.6 [2.20-2.84]	2.9 [2.71-3.05]	0.47	4.5 [4.04-5.01]	3.6 [1.36-4.27]	0.0020
SUVpeak	2.7 [2.45-2.95]	3.1 [2.84-3.59]	0.47	5.3 [4.52-6.26]	4.3 [1.45-5.24]	0.0015

Note: significance level: $P_{uncorr}<0.05$, $P_{corr}<0.0023$

SUPPLEMENTARY Fig. 1

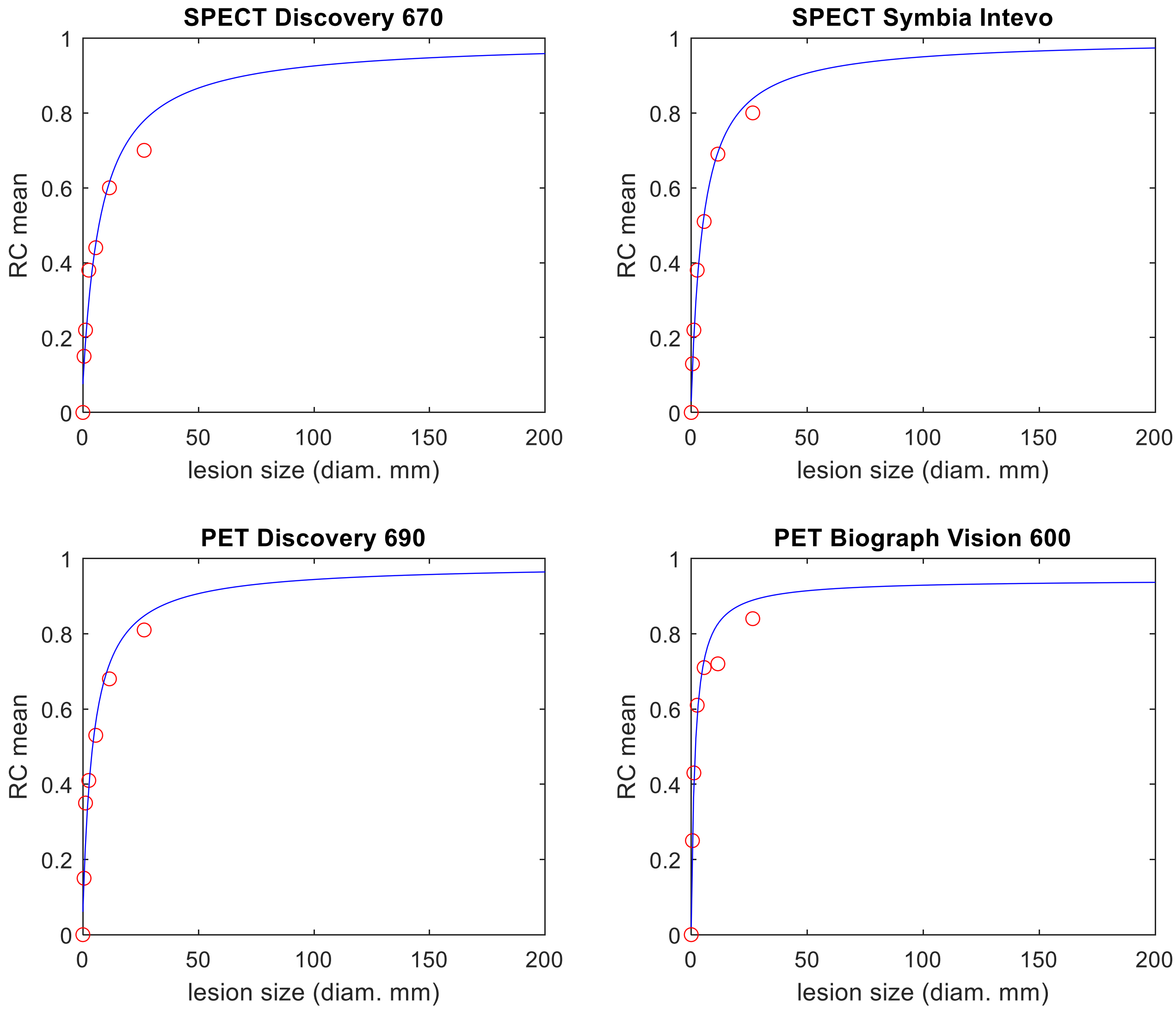

Recovery coefficients obtained from NEMA phantom experiments (red circles) and RC hyperbole fits (curves in blue) obtained for the four imaging devices involved in the study

SUPPLEMENTARY Fig. 2

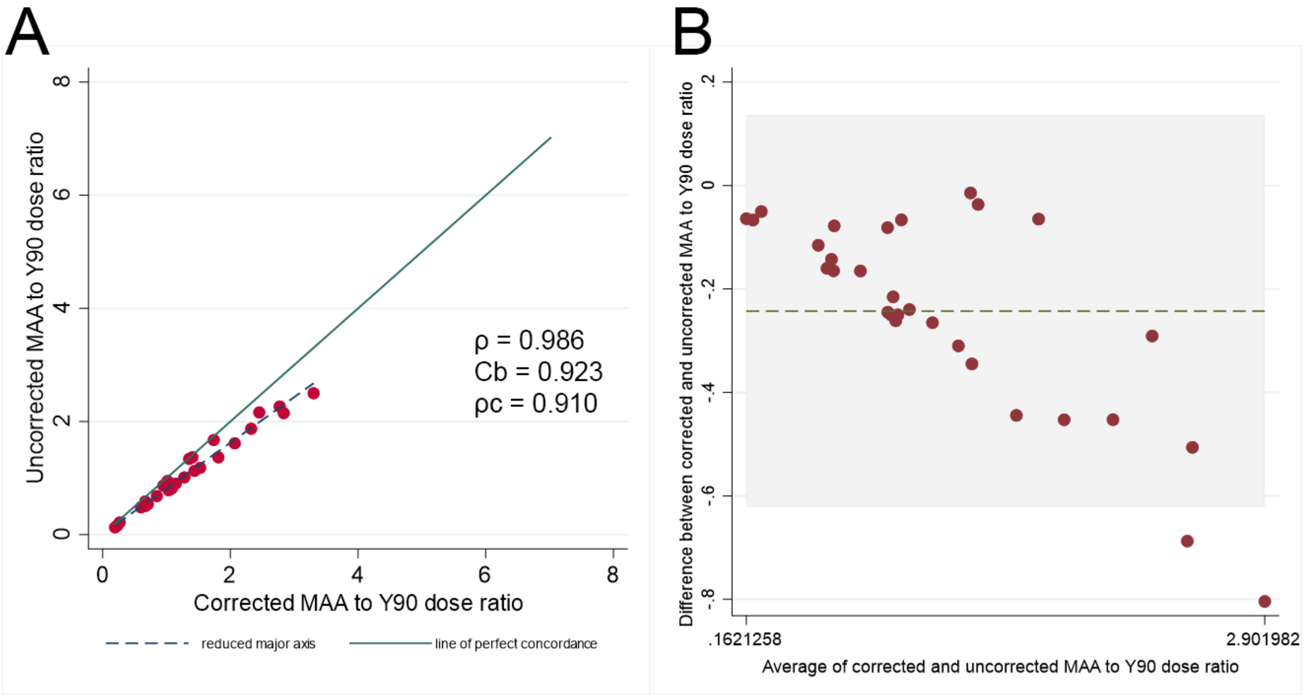

(A) Lin's concordance correlation coefficient of predicted to actual dose ratio with and without correction for the recovery coefficient. Precision (ρ) and systematic bias (Cb) were excellent. (B) Bland-Altman plot comparing agreement of predicted to actual dose ratio with and without correction for the recovery coefficient. The agreement was good with a mean difference of -0.24 (0.19 SD; 95%CI: –0.62–0.14)

SUPPLEMENTARY Fig. 3

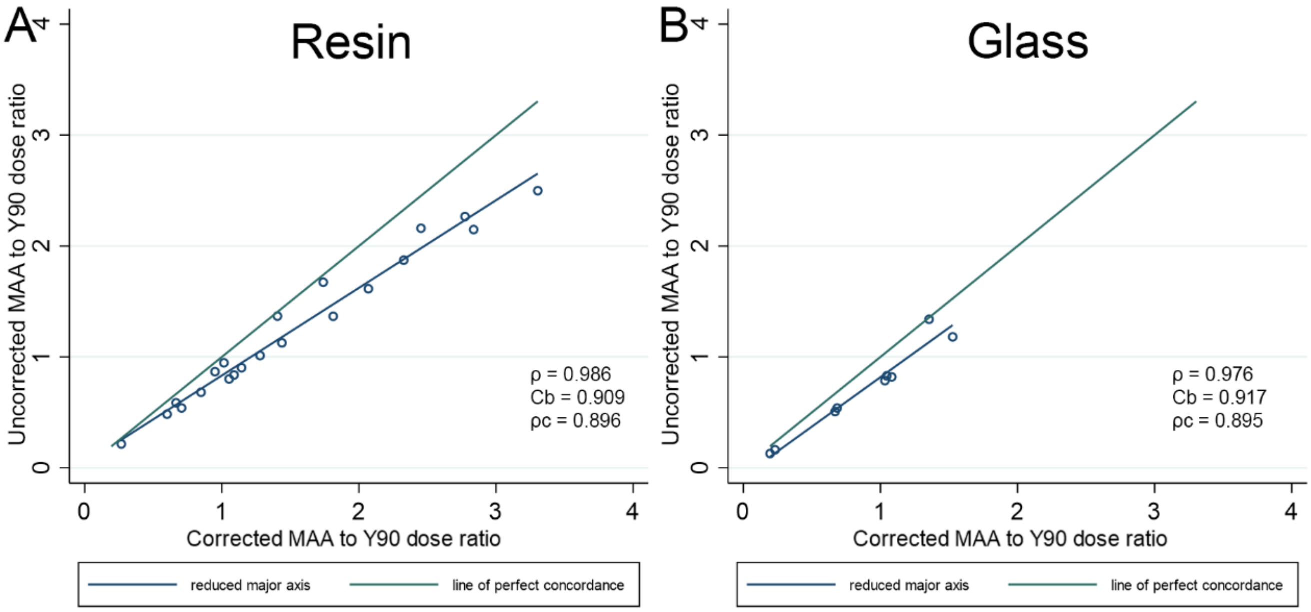

(A) Lin's concordance correlation coefficient of predicted to actual dose ratio with and without correction for the recovery coefficient for resin (A) and glass (B) microspheres. Precision (ρ) and systematic bias (C_b) were excellent
